# BrAIn: A comprehensive artificial intelligence‐based morphology analysis system for brain organoids and neuroscience

**DOI:** 10.1002/btm2.70123

**Published:** 2026-03-12

**Authors:** Burak Kahveci, Elifsu Polatli, Ali Eren Evranos, Hüseyin Güner, Gökhan Karakülah, Yalin Bastanlar, Sinan Güven

**Affiliations:** ^1^ İzmir Biomedicine and Genome Center İzmir Türkiye; ^2^ İzmir International Biomedicine and Genome Institute Dokuz Eylül University İzmir Türkiye; ^3^ Department of Molecular Biology and Genetics, Faculty of Life and Natural Science Abdullah Gül University Kayseri Türkiye; ^4^ Department of Computer Engineering, Faculty of Engineering İzmir Institute of Technology İzmir Türkiye; ^5^ Department of Medical Biology and Genetics, Faculty of Medicine Dokuz Eylül University İzmir Türkiye

**Keywords:** artificial intelligence, brain organoid, computer vision, deep learning, microfluidics

## Abstract

Human‐induced pluripotent stem cells (iPSCs) offer transformative potential for biomedical research, with iPSC‐derived organoids providing more physiologically relevant models than traditional 2D cell cultures. Among these, brain organoids (BO) are particularly valuable for drug screening, disease modeling, and investigations into molecular pathways. Accurate representation of brain morphology is critical, as more complex organoid structures better mimic the human brain. Deep learning (DL) and machine learning (ML) approaches have become integral to analyzing organoid morphology, yet tools for comprehensive, time‐resolved assessments are scarce. Here, we introduce *BrAIn*, a DL‐based application for analyzing the developmental progression of BOs. BrAIn tracks their evolution from embryoid bodies (EBs) and quantifies parameters including area, Feret diameter, perimeter, roundness, and circularity. It also classifies budding and abnormal morphologies of 3D organoids and detects monolayer neural rosette structures, key features of neuronal differentiation. Designed with accessibility in mind, BrAIn provides a no‐code interface, enabling researchers of all technical backgrounds to conduct advanced morphological analyses with ease. Our study demonstrates the application of BrAIn to evaluate the effects of different growth conditions—static, orbital shaker, and microfluidic chip‐based—on BO development. Orbital shaker cultures resulted in the largest organoids, while chip‐based systems achieved more homogeneous growth. Both conditions produced organoids with greater morphological complexity compared to static culture. BrAIn emerges as a robust, user‐friendly tool to quantify BO development and explore how versatile growth conditions influence their morphology and maturation.

## Graphical abstract

BrAIn, a deep learning‐based application for analyzing the developmental progression and morphologies of brain organoids. BrAIn tracks their evolution from embryoid bodies and quantifies parameters including area, Feret diameter, perimeter, roundness, and circularity. It also classifies budding and abnormal morphologies of 3D organoids and detects monolayer neural rosette structures.

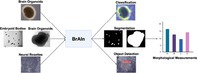

**Translational Impact Statement**

BOs are a useful model for neurodevelopmental research, disease models, and drug screening. However, their morphological complexity necessitates precise and automated analysis. We announce the BrAIn DL‐based tool for the analysis of BOs across developmental processes. By quantifying key morphological parameters and detecting critical structures like neural rosettes, BrAIn provides an accessible, no‐code platform for researchers. BrAIn analyzes BOs under various growth conditions influencing morphological complexity. Its application enhances organoid‐based studies, optimizing experimental workflows and ultimately accelerating image analysis in translational neuroscience and personalized medicine.

## 1 INTRODUCTION

Human‐induced pluripotent stem cells (iPSCs) have become one of the most promising tools for medical research, offering immense capacity for obtaining cellular phenotypes.^1^ iPSCs can also give rise to organoids, self‐formed 3D tissue mimicries, providing the structural and functional recapitulation of an organ.^2^ iPSCs‐derived brain organoid (BO) can be used in modeling neurodegenerative diseases,^3^, ^4^ drug discovery research,^5^ or platforms^6^, ^7^, ^8^, ^9^ for determination of neurotoxicity.^10^ Morphology of BOs can be a potential indicator of physiologically and functionally relevant tissue mimicry. Besides numerous advantages provided by BOs, the time and cost spent can be significant burdens even for well‐established research groups and enterprises.^11^ To improve the BO generation process, increase the yield, and minimize human‐based errors, experimental planning, optimization of resources, and interpretation of results should be well defined.^12^

Machine learning (ML) models are used in many areas such as decision support systems in medicine, analysis of medical images, disease diagnosis, and prognosis prediction.^13^ Deep learning (DL) methods provide automatic feature extraction, and they have the advantage of exploiting GPUs for parallel operations on diverse data such as image, text, sound, and genomic.^14^, ^15^ Image datasets pioneered DL methods providing diverse scenarios such as image classification, segmentation, object detection, and tracking in microscope images.^16^ Abundant and heterogeneous data assist in generation and reflect more realistic, real‐life scenarios. Images from wet lab experiments are laborious, costly, and limited in number, restraining capacity for DL models. Introducing data augmentation methods, where artificially modified inputs are generated, promise to overcome this problem providing unlimited data. Traditional data augmentation methods include operations such as flipping, cropping, rotation, and color space transformations.^17^ Traditional data augmentation methods may perform well in datasets with high data diversity^17^, ^18^, ^19^ and self‐supervised learning approaches.^20^, ^21^ However, these methods may be limited in improving model performance, especially in areas where data diversity is limited, such as medical imaging.^22^, ^23^ Generative adversarial networks^24^ can be used as an alternative to traditional data augmentation methods for data augmentation,^25^, ^26^, ^27^ especially to overcome the limitations in medical imaging datasets. Generative adversarial networks create new images based on the given datasets.^28^ StyleGAN3, an approach based on generative adversarial networks, is used in the medical field and life sciences.^29^, ^30^

DL applications in microscope images obtained from organoids can both facilitate the examination of images and provide the opportunity to optimize the experimental processes against human errors.^31^ In addition to classification, object detection and image segmentation are extensively applied in organoid analysis performed with DL.^31^ Transfer learning is a DL approach that leverages pre‐trained models to achieve better accuracy with a limited amount of data.^32^ This approach has proven to be highly effective to improve the performance measured by classification, detection, and segmentation evaluation metrics.^33^, ^34^ U‐Net^35^ and Transformer models^36^, ^37^, ^38^ are frequently used in medical image segmentation, both separately and integrated with each other.^39^, ^40^ U‐Net can effectively capture small structures with local feature extraction but cannot capture the relationship between long‐range dependencies in the global context.^40^ Transformers are more effective in the global context and have better generalization ability. Hybrid approaches using both models together are effective in both local features and global context.^39^, ^40^, ^41^

DL applications offer quite promising opportunities in the field of medical computer vision.^42^ Morphological studies on stem cells and iPSCs have been conducted using DL.^43^, ^44^, ^45^, ^46^, ^47^ Also, there are many image processing and DL‐based studies for use in the morphological analysis of organoids.^31^ These studies can be divided into three main areas: classification,^48^, ^49^, ^50^, ^51^, ^52^, ^53^ segmentation,^54^, ^55^, ^56^, ^57^, ^58^, ^59^, ^60^, ^61^, ^62^, ^63^, ^64^, ^65^ and detection.^66^, ^67^, ^68^ There are a few studies on the classification^69^, ^70^, ^71^ and segmentation^70^, ^72^, ^73^, ^74^ based morphological analysis of neural organoids. Neural organoid studies using ML and DL models are presented in Table 1.

**TABLE 1:** Classification and segmentation studies using neural organoids.

	Dataset	Method	References
Classification	Midbrain organoids	Random forest	^69^
	Neural organoids	Convolutional neural networks	^71^
Segmentation	Cerebral organoids	U‐Net	^70^, ^72^
	Brain organoids	U‐Net, K‐means, support vector machines	^73^
	Brain organoids	Mu‐Net	^74^

Organoid morphology analysis and developmental morphology monitoring are quite critical. Although semi‐automated microscope applications provide a certain level of analysis, they are still time‐consuming. In addition, since human intervention is required, there may be potential incorrect measurements and errors. As summarized in Table 1, there are studies that perform organoid morphology analysis. However, there is no comprehensive tool that can analyze the entire developmental process. In this study, the BrAIn tool was developed to analyze the developmental process of BOs. Morphological analyses of the developmental process of the BO can be analyzed and quantified with BrAIn under three different sections.

- In the classification section, three different classes of morphologies (normal, abnormal, and budding) that may occur during the process of creating BOs from embryoid bodies (EBs) can be classified with BrAIn.
- BrAIn can segment EBs and BOs with high precision, and the developmental processes of BOs can be quantified with different parameters. Developmental processes were examined under different growth conditions. The different effects of these conditions on growth and morphology were analyzed with BrAIn.
- In addition, neural rosette structures were identified, which are quite important in the process of generating iPSC‐derived neurons and are difficult to detect with the naked eye.

With BrAIn, every step of the BO generation process is automated, minimizing human‐based errors, saving time and decreasing cost by planning the experimental processes based on artificial intelligence, all through a user‐friendly interface that even users with no coding knowledge can easily navigate.

## 2 MATERIALS AND METHODS

### 2.1 hiPSCs culture

Human iPSCs are kindly provided by Prof. Tamer Onder.^75^ hiPSCs were cultured in 6‐well plates coated with 1% embryonic stem cell (ESC) qualified Matrigel (Corning) with mTeSR1 (STEMCELL Technologies) medium. Culture was maintained with a daily change of fresh medium until cells reached 80% confluency. hiPSCs were passaged with ReLeSR (STEMCELL Technologies) at a ratio of 1:10. iPSCs culture and organoid development were carried out at 37°C under 5% carbon dioxide conditions.

### 2.2 Neuronal differentiation

hiPSCs were differentiated into neural progenitor cells (NPCs) according to the previously published protocol^76^ using dual SMAD inhibition. Briefly, iPSCs detached with ReleSR and seeded on low attachment 6‐well plates in human embryonic stem cell medium (hESCM) (DMEM/F‐12) (Gibco, Thermo Fisher Scientific), 20% knockout serum replacement (KOSR) (Gibco, Thermo Fisher Scientific), 1% non‐essential amino acids (NEAA) (Lonza), 1% non‐essential amino acids (NEAA) (Lonza), 1% GlutaMAX supplement (Gibco, Thermo Fisher Scientific), 1% penicillin/streptomycin, 100 mM 2‐mercaptoethanol with the fresh addition of ROCKi (50 μM) for 2 days. After 2 days, medium was replaced with SMAD inhibitors using 5 μM SB 431542 and 10 nM LDN 193189. EBs were incubated for a further 2 days. At day 4, medium was replaced with Neural induction medium (NIM) (DMEM/F‐12, 1% N2, 1% NEAA, 1% penicillin/streptomycin, and 2 μg/mL heparin). EBs were incubated as suspension for 2 days, then seeded on growth factor‐reduced Matrigel (Corning) coated wells with the NIM for the rosette formation. Media were changed every 2 days until neural rosette structures appeared.

### 2.3 Development of brain organoids

hiPSCs were differentiated into BOs according to a previously published protocol.^6^ Briefly, hiPSCs were removed from Matrigel with ReleSR and dissociated by pipetting gently. Cells were seeded on low attachment U‐bottom 96‐well plates at a density of 9 × 10^3^ cells/well with embryonic stem cell medium (hESCM) with the addition of ROCKi (50 μM) and 4 ng/mL bFGF.

**FIGURE 1:**
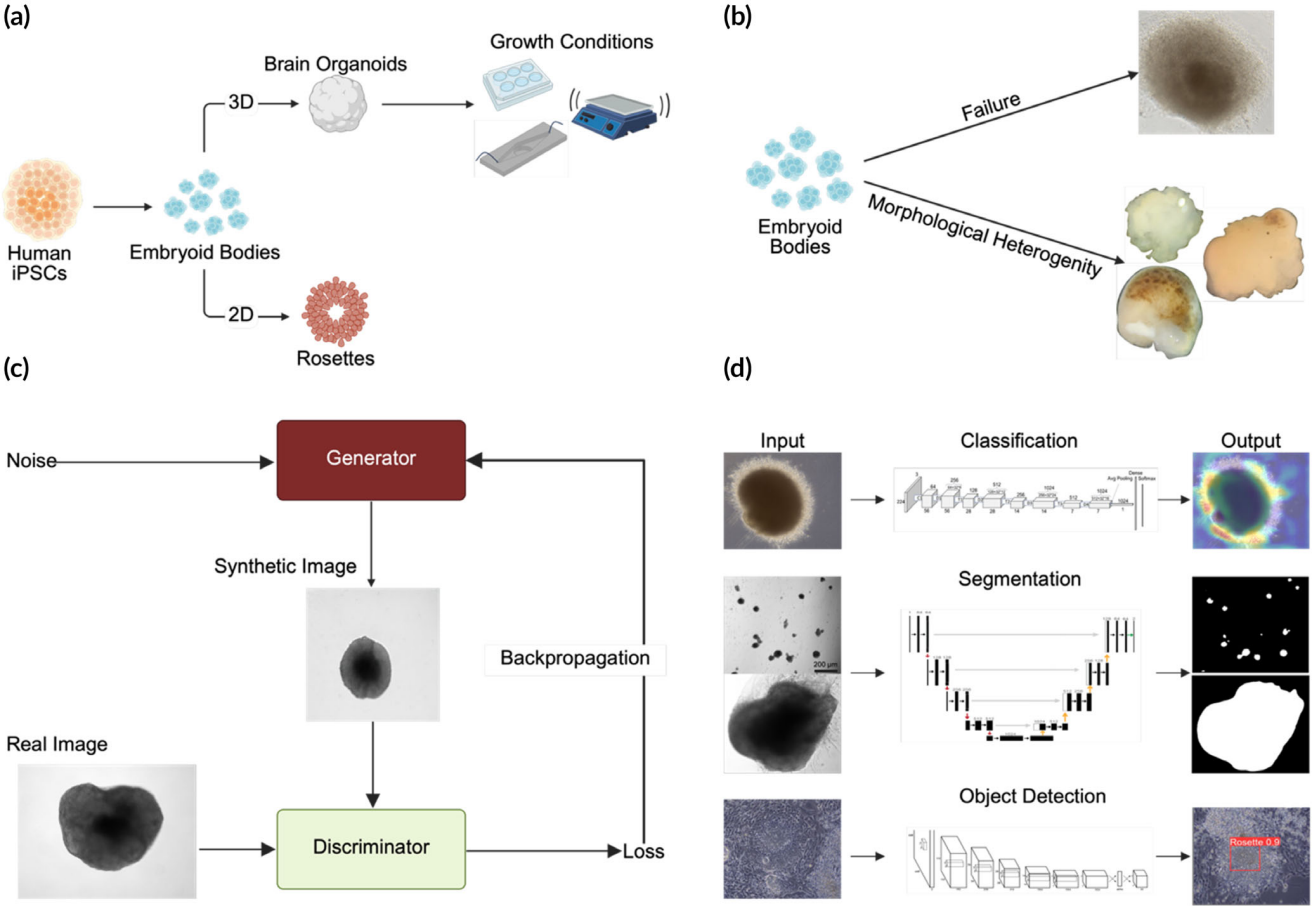
Workflow summarizing experimental and computational stages of BrAIn. (a) Schematic illustration showing generation of BOs and Rosettes derived from hiPSCs. (b) BOs originate from the same EB stage but have high heterogeneity. (c) Schematic diagram of the StyleGAN3 model used in data augmentation to ensure better results and generalization of BrAIn. (d) Illustration of the DL models used for the three sections included in BrAIn, classification, segmentation and object detection.

Cells were incubated for 6 days, with 50% medium replacement performed every other day. EBs were transferred into low attachment 24‐well plates with Brain organoid Neural Induction Media (BONIM) (DMEM/F‐12, 0.01% N2, 1% Glutamax, 1% penicillin/streptomycin, and 2 μg/mL heparin) for 4 days. EBs were embedded into basement Matrigel (Corning) with Cerebral Organoid Differentiation Media (CODM) without the addition of vitamin A (1:1 DMEM/F12) and Neurobasal Medium (Gibco, Thermo Fisher Scientific, 0.5X N2, 1X GlutaMAX, 0.5X NEAA, 1X B27 w/o VitA, 0.25 (v/v) insulin, %0.02 (v/v) 2‐mercaptoethanol). After 4 days, immature organoids were transferred into microfluidic chips, orbital shaker, and static conditions according to their groups with the addition of vitamin A into CODM. Cells were incubated for 48 days with medium changes 2–3 times a week (Figure 1a, Figure S1).

### 2.4 Fabrication of microfluidic chips

Microfluidic chips consist of multilayer poly(methyl methacrylate) (PMMA). Channels were designed by AutoCAD (Autodesk) and CorelDRAW (Corel Co.) (Figure S1). PMMA layers were cut with a laser cutter (Epilog‐MINI) and attached by double‐sided adhesive. Sterilization of microfluidic chips was performed with 70% ethanol following UV exposure for 30 min. BOs were cultured in the microfluidic chip with 35 μL/min.

### 2.5 Immunofluorescence staining

EBs and BOs were fixed with 4% paraformaldehyde (Sigma‐Aldrich) for 24 h at 4°C. BOs were embedded in cryomatrix (OCT, Fisher Healthcare) and cryosections were taken with Leica CM1950 (Leica Biosystems, Germany). EBs and BOs were permeabilized with permeabilization buffer (0.3% Triton X‐100 [neoFroxx, GmbH, Cat no. 8500] [v/v] in PBS [Gibco, Thermo Fisher Scientific, Cat no. P04‐36500]) for 15 min at room temperature. Cells were treated with blocking buffer (0.05% Tween 20 [Sigma‐Aldrich, Cat no. P7949] [v/v], 1% Bovine Serum Albumin [BSA; Sigma‐Aldrich, Cat no. A2153] [w/v] in PBS) for 1 h at room temperature. Primary antibodies (Table S1) were added to the relevant samples and incubated overnight at 4°C. Cells were stained with relevant secondary antibodies for 2 h at room temperature. Nuclei were stained with 4,6‐diamidino‐2‐phenylindole (0.5 μg/mL) (DAPI; Neofroxx, Cat no. 1322). Images of the samples were captured with a fluorescence microscope (Olympus IX71) and a confocal microscope (Zeiss LSM880).

### 2.6 Datasets and training of models

In the training process of BrAIn, five datasets were used: two datasets containing normal‐abnormal classes and normal‐budding classes for the classification section (Figure 1b), time‐dependent BO and EB datasets for the segmentation section, and a rosette dataset for the object detection section.

Details of the dataset and model information are given in Table 3. In addition, the BOs dataset in the segmentation section and the rosette datasets in the object detection section were augmented using the StyleGAN3 model.^77^ The working process of the Generative Adversarial Network on which StyleGAN3 is based is given in Figure 1c. As summarized in Figure 1d, BrAIn contains three different parts: classification, segmentation, and object detection. Transfer learning and a fivefold cross‐validation approach were used in classification. In this section, 5 models were used: DenseNet,^78^ Inception,^79^ ResNet50,^80^ VGG16^81^ and Xception.^82^ In the segmentation part, the U‐Net^35^ model was used for the segmentation of BOs and EBs with a 5‐fold cross‐validation approach. In the object detection part, the YOLOv8^83^ model was used to detect rosette structures from 2D cell culture images.

In classification model training step, the batch size was determined as 32 and the resolution of the images was 550 × 550. Early stopping approach was used throughout the training. Training was terminated if no improvement was observed in the validation loss value for three epochs. In the segmentation part, U‐Net was used for the segmentation of BOs and EBs. Datasets for U‐net were prepared with OrganoLabeler developed by our group.^84^ In the models, the batch size was determined as 4 and the learning rate value was used as 1e‐3. Cross validation approach was applied during the training process of the models and the fold value was determined as 5. Two BOs developed from static, microfluidic chip and orbital shaker systems were used for morphology analysis. In the object detection section, the YOLOv8 model was used to detect neural rosettes. In model training, the batch size was determined as 8 and the initial learning rate was used as 0.01. The training processes and parameter details of the models are given in Table 2. All datasets used were captured from an inverted microscope. For statistical analysis, Python programming language (3.13.3) scipy, stats, statsmodels libraries were used. Plots were generated using seaborn and matplotlib libraries.

**TABLE 2:** Hyperparameters and model architectures for BrAIn models.

Component	U‐Net (segmentation)	YOLOv8 (object detection)	Classification models^a^
Framework	TensorFlow / Keras	Ultralytics YOLOv8 (PyTorch)	TensorFlow/Keras
Input size	256 × 256 (grayscale)	700 × 700 (RGB)	550 × 550 × 3
Model depth	4 encoder‐decoder levels	YOLOv8n (nano) backbone with PAN neck	ImageNet‐pretrained
Initial filters	64	CSP‐based convolutional layers	
Activation	ReLU (hidden layers), sigmoid (output layer)	SiLU (Swish)	Softmax
Normalization	Batch normalization (after each Conv layer)	Batch normalization	—
Optimizer	Adam	SGD with momentum	Adam
Learning rate	1e‐3	0.01 (with cosine LR scheduler)	1e‐6 (EarlyStopping, ReduceLROnPlateau)
Loss function	Binary crossentropy	CIoU loss (bbox), Objectness + Class loss	Categorical crossentropy
Batch size	4	8	32
Epochs	10	100	50

^a^ DenseNet121, Inception, ResNet50, VGG16, Xception.

### 2.7 Model architecture and Hyperparameters

ImageNet‐pretrained models were used for the classification section. Softmax was preferred as the activation function and early stopping approach was applied. U‐Net model has a depth of 4 encoders and 4 decoders and 1024 bottleneck filters. Each block contains two convolutional layers with a kernel size of 3 × 3 and ReLU activation. Batch Normalization is used for improved convergence for all layers. Images are taken as input with a resolution of 256 × 256 and their segmented form with sigmoid activation is given as mask. Binary cross‐entropy is used as loss function and batch size is given as 4 (Table 2). We chose yolov8m as the YOLOv8 model. We used a batch value of 8 and an image resolution of 700 × 700. This model includes a CSPDarknet backbone and an anchor‐free detection head, and the SGD optimizer was used during training (Table 2).

### 2.8 Ethics statement

This study has been approved by the Institutional Review Board of IBG, protocol number 2024‐007.

## 3 RESULTS

### 3.1 Characterization of neural rosettes and brain organoids

**FIGURE 2:**
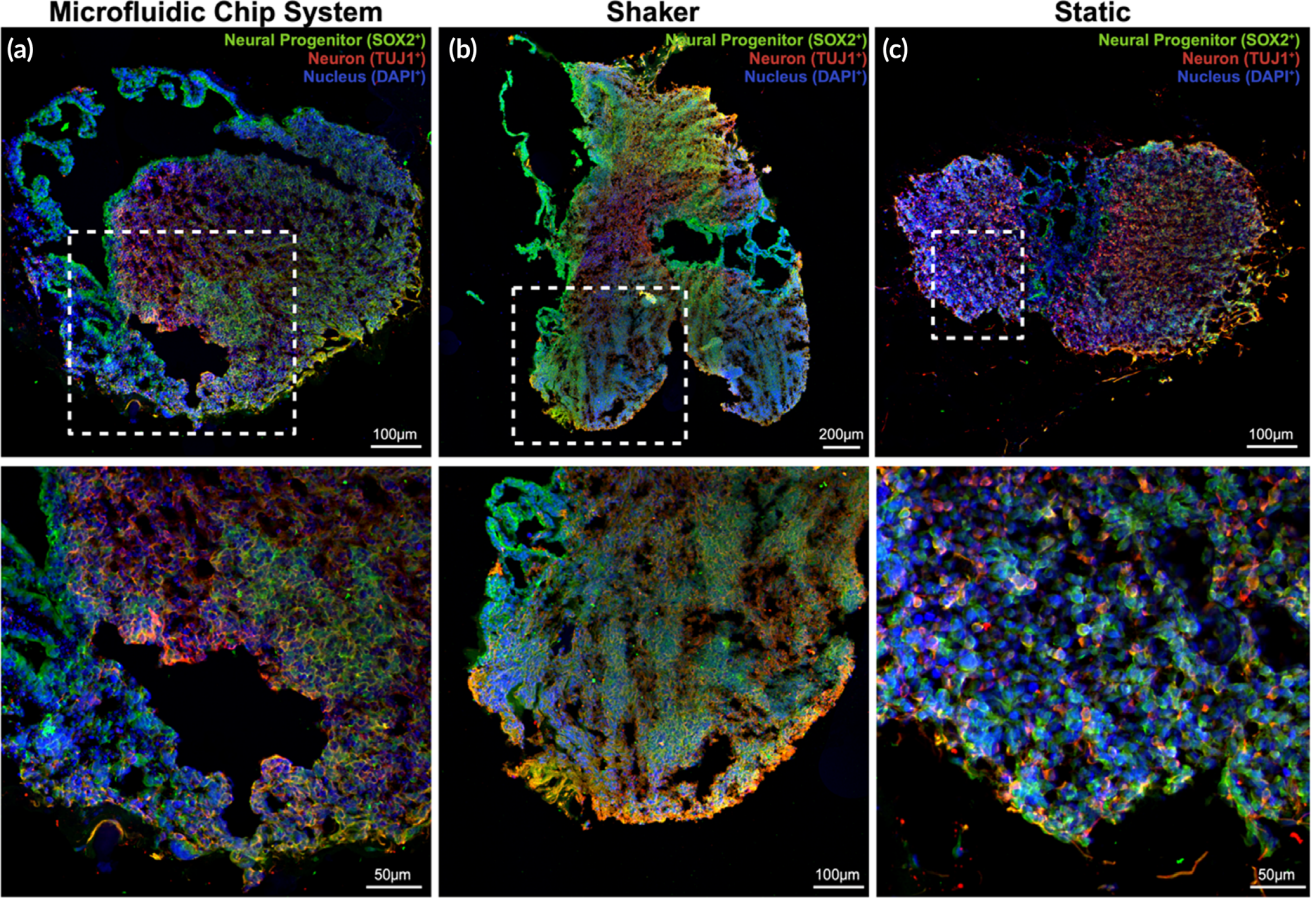
Immunofluorescence characterization of Day 30 BOs demonstrating SOX2 (green) expressing neural progenitor cells and TUJ1 (red) positive neurons. Cell nuclei were stained with DAPI (blue). (a) BOs grown in a microfluidic chip system. (b) Organoids cultured in an orbital shaker. (c) BOs under static conditions. The lower panel provides a higher magnification view of the highlighted area.

BOs have previously been established under dynamic conditions, such as orbital shaker and microfluidic chip systems, alongside traditional static growth methods. To comprehensively evaluate the performance of BrAIn across diverse culture conditions, BOs were generated in three distinct environments: microfluidic chip, orbital shaker, and static culture. Phenotypic characterization of BOs was performed with immunofluorescence staining, validating their differentiation potential through demonstrating presence of neural progenitor (SOX2) and neuronal cells (TUJ1) (Figure 2a–c).

EBs mimic early embryogenesis and have the potential to differentiate into the three germ layers, notably the ectoderm layer, which gives rise to the brain tissue. In this study, we generated EBs from hiPSCs using a 6‐day suspension culture and confirmed their trilineage differentiation potential into ectoderm, endoderm, and mesoderm (Figure S2). Neural rosettes, formed during ectodermal differentiation, contain NPCs capable of differentiating into neurons, oligodendrocytes, and astrocytes—the primary neuroectodermal cell types. To enable BrAIn to accurately detect neural rosettes, which are challenging to identify through visual inspection, we developed hiPSC‐derived neural rosettes and validated their structural integrity and differentiation potential using immunofluorescence staining (Figure S2).

### 3.2 Classification of brain organoid morphologies

**FIGURE 3:**
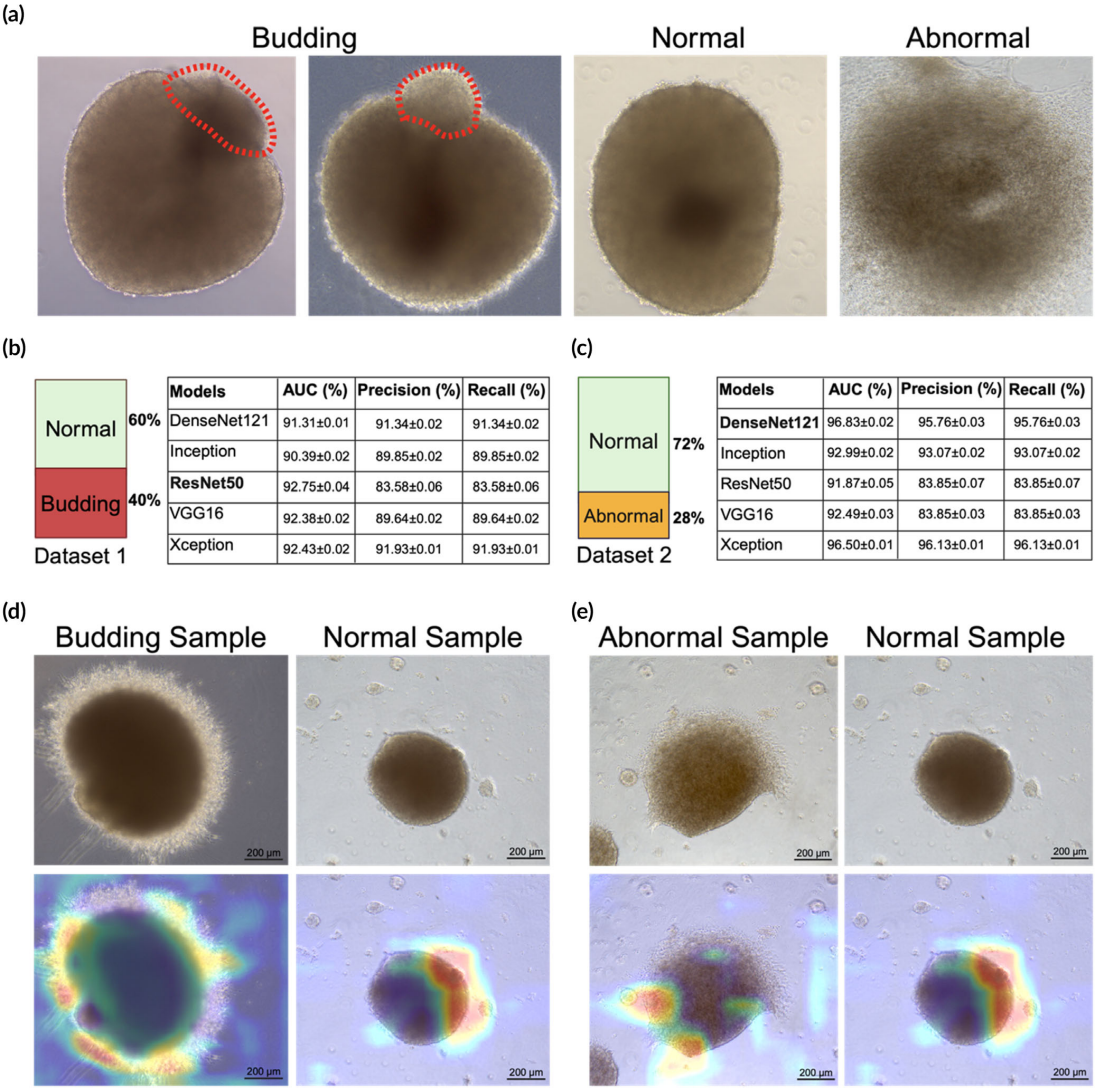
BrAIn's classification module for BOs. (a) Morphological features used for classification tasks, including normal, budding, and abnormal categories. (b) Class distribution and model performance metrics for normal versus budding classification. (c) Class distribution and model performance metrics for normal versus abnormal classification. (d) Grad‐CAM heatmap illustrating the feature distribution utilized by the model for normal versus budding classification. (e) Grad‐CAM heatmap illustrating the feature distribution utilized by the model for normal versus abnormal classification. The red regions indicate high importance, highlighting the key areas used by the model during the classification process.

Morphology plays a crucial role in determining the quality of BOs during their transition from EBs to organoids. Perturbation of organoid morphology at early‐stage formation of budding structures serves as key indicators of successful BO differentiation. The classification module of BrAIn categorizes organoids into three distinct classes: normal, which lacks budding yet is considered acceptable; budding, characterized by the presence of perturbated zones; and abnormal, where cells fail to establish the basic structural integrity required for proper EB formation (Figure 3a). BrAIn focuses on two primary classification tasks: distinguishing normal from abnormal organoids and differentiating between normal and budding organoids.

In the normal‐budding classification task, the dataset consisted of 60% normal and 40% budding samples. Among the tested models, ResNet50 achieved the highest performance, with AUC, precision, and recall values of 92.75%, 83.58%, and 83.58%, respectively (Figure 3b). For the normal‐abnormal classification task, which had a class distribution of 72% normal and 28% abnormal samples, DenseNet121 outperformed other models with an AUC of 96.83%, precision of 95.76%, and recall of 95.76% (Figure 3c). Grad‐CAM heatmaps were employed to visualize the regions most influential in the model's decision‐making process, with red areas indicating the most significant features used for classification (Figure 3d,e).^85^

### 3.3 Brain organoid segmentation and morphological evaluation

**FIGURE 4:**
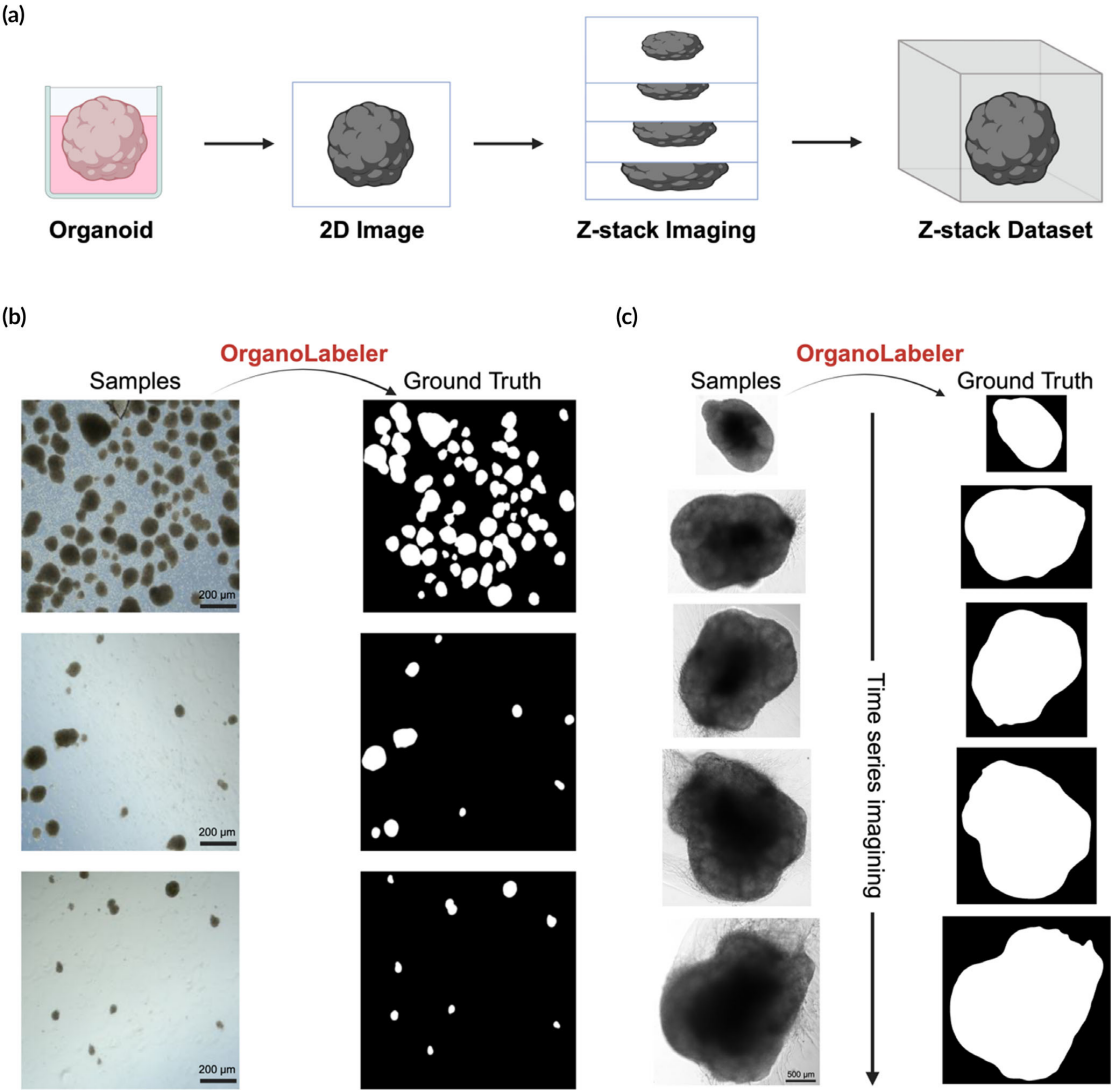
Dataset generation workflow for BrAIn. (a) Z‐stack imaging methodology used to accurately capture the three‐dimensional complexity of BOs. (b) Automated annotation of the EB dataset using the OrganoLabeler tool. (c) Time‐series image acquisition for the developmental analysis of BOs, with dataset annotation performed using OrganoLabeler.

To accurately capture the three‐dimensional structure of BOs, the z‐stack imaging method was employed during dataset creation, with a layer spacing of 10 μm (Figure 4a). OrganoLabeler, a labeling tool developed by our group, was utilized to annotate both the EB and BO datasets (Figure 4b,c). Throughout the EB formation process, a segmentation model was developed based on images collected over 4 days, allowing for a detailed analysis of morphological changes. To further assess BO development, images were captured at 7‐day intervals from days 20 to 62, providing comprehensive insights into organoid growth dynamics.

Beyond the data used for training and internal testing (Table 3), an independent “analysis dataset” was specifically curated for both brain organoid (BO) and embryoid body (EB) segmentation tasks. These analysis datasets, consisting of 126 BO images and 210 EB images, were used to provide an unbiased assessment of model generalization capabilities on truly unseen data.

**TABLE 3:** Dataset and model information for BrAIn.

Model	Dataset name	Section	Train and test data distribution	Epoch	Batch size	Loss
ResNet50	Budding‐normal	Classification	265–67	50	16	Categorical cross entropy
DenseNet121	Abnormal‐normal	Classification	188–52	50	32	Categorical cross entropy
U‐Net	Embryoid body	Segmentation	132–33	20	4	Binary cross‐entropy
U‐Net	Brain organoid	Segmentation	1273–318 (898 synthetic images)	10	4	Binary cross‐entropy
YOLOv8	Neural rosette	Object detection	338–35 (293 synthetic images)	100	8	Distribution focal loss

U‐Net models were trained using the parameters given in Table 2. The model trained with the EB dataset achieved a mean Intersection over Union (IoU) of 96.14 ± 0.022% and a mean Dice coefficient of 97.72 ± 0.002% on the test set. Regarding the BO dataset, the initial U‐Net model yielded an IoU of 73.17 ± 0.003% and a Dice coefficient of 77.55 ± 0.005%. To enhance model performance, a total of 898 synthetic BO images were generated using the StyleGAN3 model, followed by data augmentation (Figure S4). The biological relevance of the produced synthetic BO images was consistent in terms of morphological features when compared with real BO images (Table S2, Figure S4). As a result, the model trained with the augmented dataset achieved an IoU of 98.85 ± 0.008% and a Dice coefficient of 98.58 ± 0.007%.

**FIGURE 5:**
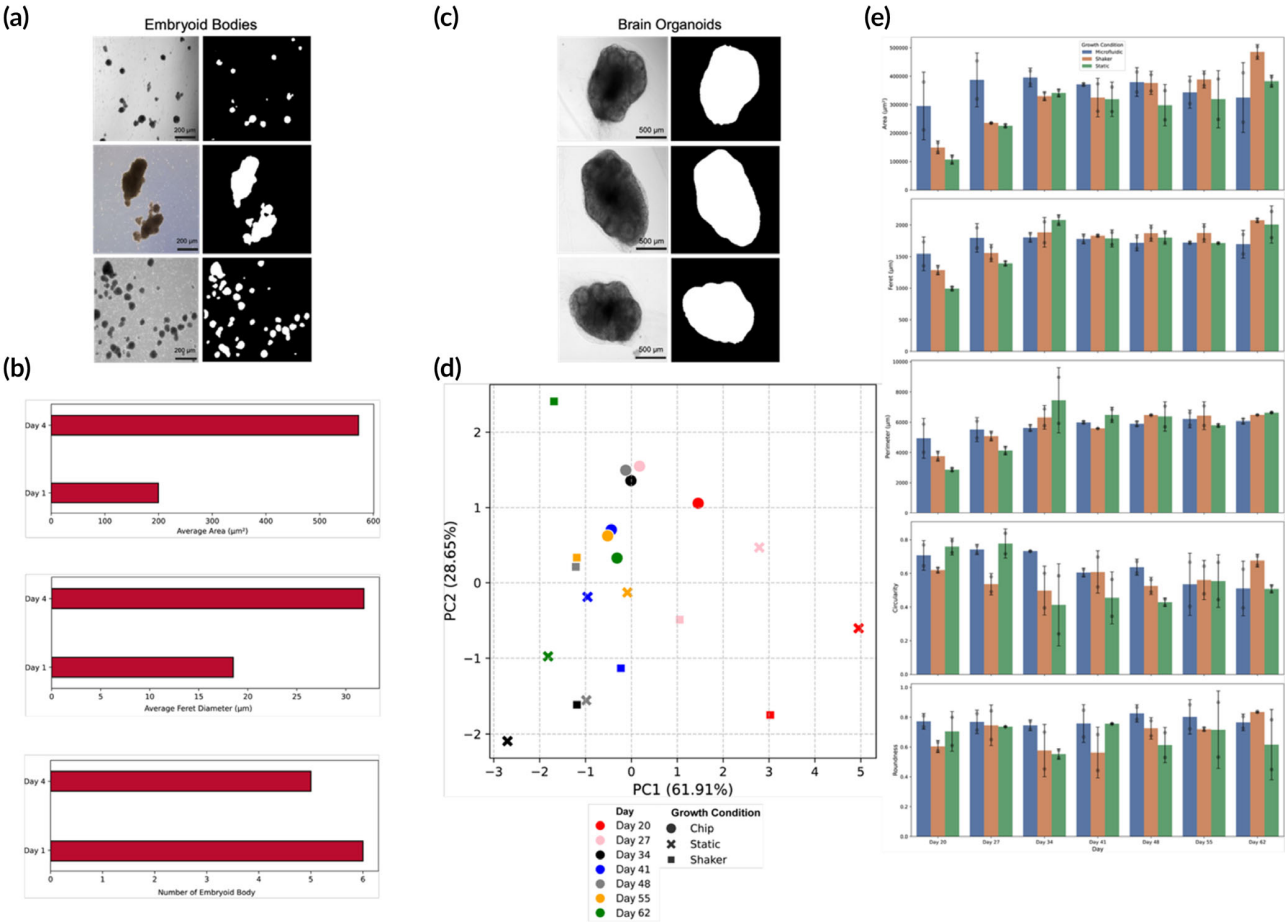
Time‐series segmentation and morphological analysis of BOs using BrAIn. (a) Representative images of EBs before and after segmentation using BrAIn. (b) Quantification of EB number, average area, and average Feret diameter over a 4‐day period in a six‐well plate format. (c) Representative raw and segmented images of BOs obtained from the BrAIn analysis pipeline. (d) Principal component analysis (PCA) plot generated based on morphological parameters, including area, Feret diameter, perimeter, circularity, and roundness, to illustrate variance across different growth conditions and time points. (e) Bar plots depicting the temporal progression of area, Feret diameter, perimeter, circularity, and roundness values of BOs under static, orbital shaker, and microfluidic chip‐based growth conditions.

The EB analysis dataset was generated using images captured on days 1 and 4 from the same well of a six‐well plate (Figure 5a, Figure S5D). Morphological analyses were performed by evaluating area, Feret diameter, and the number of EBs (Figure 5b). A decrease in the average EB number on day 4, along with an increase in the average area and Feret diameter, suggests that some EBs may have merged over time to form larger structures. The average area increased from 199.65 μm^2^ on day 1 to 572.28 μm^2^ on day 4 (Figure 5b). Similarly, the Feret diameter showed an increase from 18.51 to 31.84 μm. Following the segmentation step (Figure 5c) described above, roundness, circularity, area, Feret diameter, and perimeter measurements (Figure S5) were obtained for the time‐dependent developmental morphological analysis of BOs using the BO analysis dataset. Principal component analysis (PCA) based on time points and growth conditions was performed using the extracted measurement data (Figure 5d). The largest variance in the dataset was observed in PC1, represented along the x‐axis. As shown in Figure S5B, the parameters that contributed most to the variance in PC1 were area, perimeter, and Feret diameter, exhibiting a negative correlation. The PCA plot further highlights the time‐dependent morphological changes in BO development. In the early stages (Days 20 and 27), BOs across all growth conditions were clustered on the right side of the plot, while, as maturation progressed, clustering shifted toward the left. When evaluated in terms of growth conditions, organoids cultured in the microfluidic chip system formed a distinct cluster on the positive side of PC2, primarily influenced by roundness and circularity (Figure S5).

The time‐dependent development of BOs under different growth environments was analyzed in terms of area changes using BrAIn (Figure 5e). The results indicate that the orbital shaker condition consistently promoted positive area growth dynamics. In contrast, in the microfluidic chip‐based growth condition, initial positive growth stabilized around day 34, after which an aerial decline was observed. Although growth progressed positively under static conditions, it remained lower compared to the orbital shaker condition.

The Feret diameter was also evaluated to assess the time‐dependent development dynamics of BOs under different growth conditions (Figure 5e). The orbital shaker condition was the only environment that exhibited regular growth with minimal heterogeneity. While organoids cultured under static conditions occasionally reached values comparable to those in the orbital shaker condition, they failed to exhibit homogeneous growth patterns. On the other hand, organoids grown in the microfluidic chip system, despite achieving comparable maximum diameters, did not show sustained positive development over time. During the early stages (days 20–34), Feret diameter values were higher under microfluidic chip system and orbital shaker conditions but lower in static conditions. Although static condition organoids outperformed the other conditions by the end of the early phase, they could not sustain these levels in the later stages (days 48–62).

The perimeter changes of BOs under different growth conditions were also analyzed using BrAIn (Figure 5e). No significant differences were observed in perimeter measurements across all three growth conditions throughout the developmental process. Furthermore, no major effects of growth conditions were detected in the early and late developmental stages. BO development was assessed by analyzing the effects of different growth conditions on circularity and roundness values (Figure 5e).

Circularity analysis revealed that BOs exhibited a more circular morphology across all growth conditions during the early stage. In the maturation phase, morphological heterogeneity increased gradually in the microfluidic chip system, whereas a sharper increase was observed in the orbital shaker and static conditions. In terms of roundness, BOs that initially exhibited a more heterogeneous morphology under orbital shaker conditions transitioned to a more homogeneous morphology in the microfluidic chip and orbital shaker systems as they matured, while increased heterogeneity was observed in static conditions. To evaluate growth conditions on brain organoid morphology, we performed a one‐way ANOVA test on circularity and roundness after verifying data normality and variance homogeneity. While the analysis showed that growth conditions did not have a significant effect on circularity (*p* = 0.360), the effect on roundness was statistically significant (*p* = 0.032) (Figure S5). Post‐hoc Tukey HSD tests of the microfluidic chip system and the static condition affected the roundness statistically significantly (*p* = 0.0424). The difference between the microfluidic chip system and the shaker condition was not statistically significant (*p* = 0.070), but close to the threshold.

**FIGURE 6:**
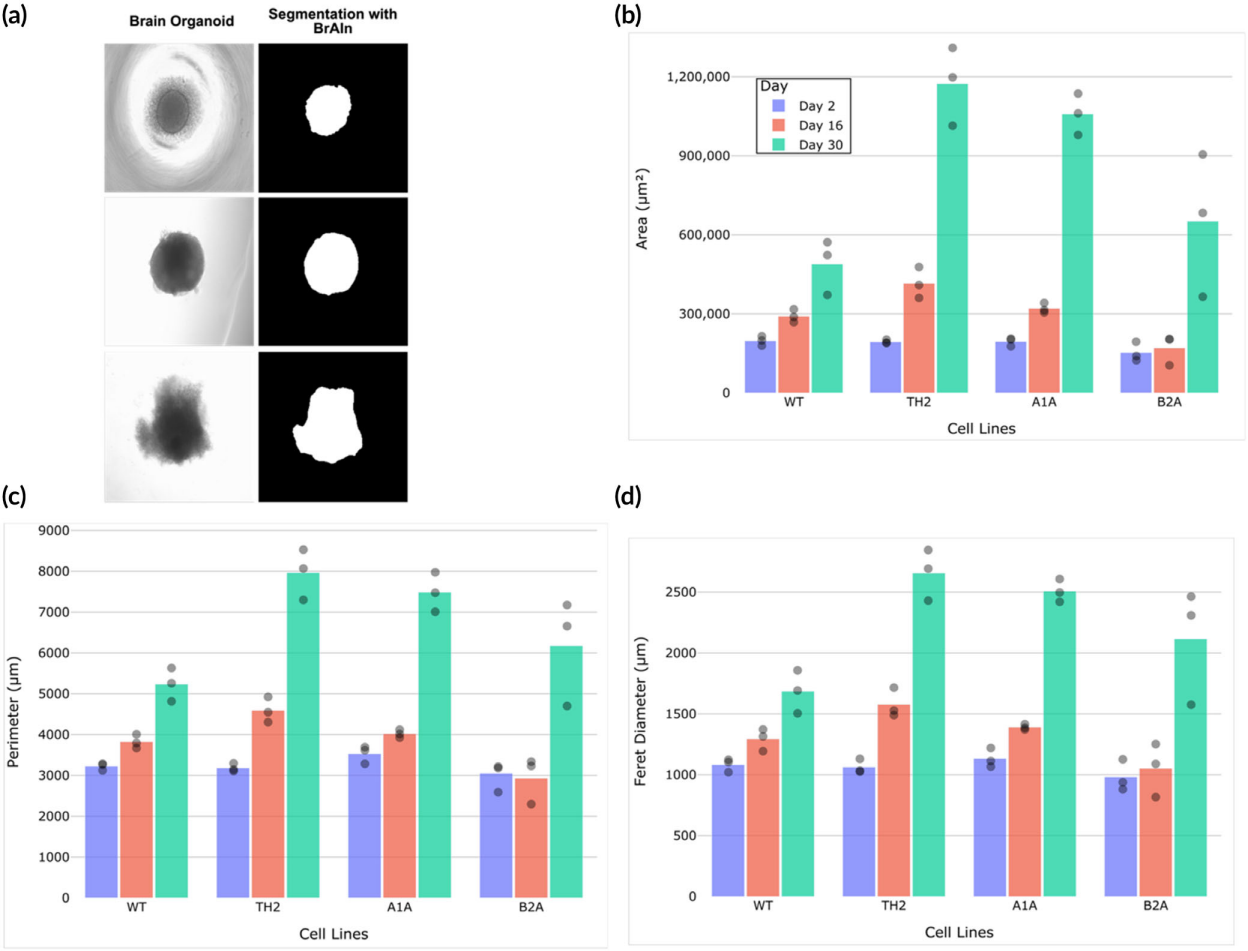
Segmentation and measurements of BOs generated using four different cell lines (WT, TH2, A1A, and B2A) by BrAIn. (a) Representative raw and segmented images of BOs generated from the WT cell line (healthy control). (b) Time‐dependent changes in organoid area across different cell lines. (c) Cell line‐specific time‐dependent perimeter changes of BOs. (d) Time‐dependent variations in Feret diameter across different cell lines.

BrAIn was further validated using the BO dataset generated by Schröter et al.^86^ The segmentation performed with BrAIn achieved a Dice score of 84%, demonstrating its reliability. This dataset included BOs derived from four different cell lines—one healthy control and three patient‐derived lines. These results indicate that BrAIn successfully analyzed an independent BO dataset with acceptable accuracy, and the morphological analysis results were consistent with the published findings.^86^ Time‐dependent morphological analysis (days 2, 16, and 30) of three randomly selected organoids from each group was performed using BrAIn (Figure 6a, Figure S6).

Upon examining area changes, although the healthy control cell line exhibited a consistent increase over time, it lagged behind organoids derived from patient cell lines by day 30. All patient‐derived cell lines demonstrated a general trend of positive growth, with organoids generated from the TH2 cell line achieving the largest area (Figure 6b). A similar trend was observed for perimeter and Feret diameter, where all cell lines showed overall growth progression (Figure 6c,d). Notably, TH2 organoids displayed the highest values on day 30, whereas B2A organoids followed a developmental trajectory similar to the healthy control cell line.

Roundness and circularity analyses revealed minimal variations among organoids generated from the four cell lines (Figure S6b). However, organoids derived from the A1A cell line exhibited a more heterogeneous morphology compared to the others. Conversely, organoids generated from B2A and TH2 cell lines initially exhibited homogeneous morphology but showed increased heterogeneity over time. In contrast, the morphology of organoids generated from the healthy control cell line remained relatively homogeneous (Figure S6b). Circularity analysis indicated initial heterogeneity in the B2A and A1A cell lines, which gradually attained a more homogeneous morphology, whereas the remaining cell lines maintained their homogeneous structure throughout the maturation process (Figure S6b).

To evaluate the performance of BrAIn, we compared it with two organoid morphology analysis tools, OrganoID^56^ and OrgaExtractor,^57^ and a foundation segmentation model, segment anything (SAM)^87^ for the BO dataset (Figure S8). OrganoID achieved a segmentation performance with a mean Intersection over Union (IoU) of 35% and a Dice coefficient of 46%, whereas OrgaExtractor yielded significantly lower scores, with an IoU of 2% and a Dice coefficient of 4%. SAM achieved the best result among the three tools with 76.80% IoU and 85.42% Dice coefficient score. Additionally, these tools were evaluated using our EB dataset (Figure S9). In this dataset, OrganoID achieved an IoU of 44% and a Dice coefficient of 58%, while OrgaExtractor exhibited considerably lower performance, with an IoU of 5% and a Dice coefficient of 9%. SAM achieved 31.07% IoU and 40.74% Dice coefficient score. These results demonstrate that BrAIn provides significantly higher segmentation accuracy compared to existing tools, further underscoring its robustness and reliability across different organoid datasets.

### 3.4 Neural rosette detection

**FIGURE 7:**
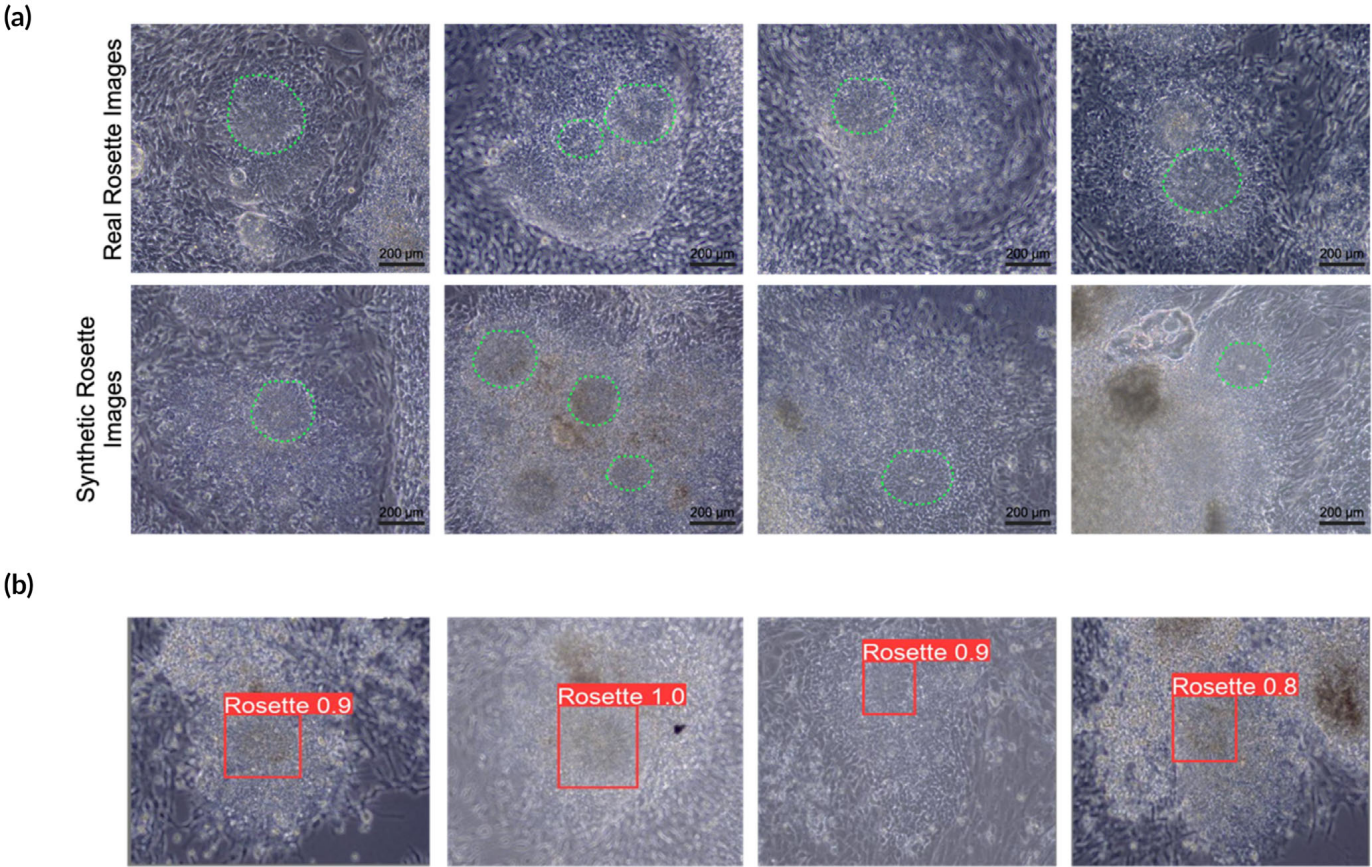
Detection of neural rosettes from iPSC‐derived 2D neural culture images. (a) Real neural rosette images alongside synthetic neural rosette images generated using a DL model. Green dashed lines indicate identified neural rosettes. (b) BrAIn's detection results for neural rosettes in 2D neural culture images.

Neural rosettes, critical structures in iPSC‐derived neuron generation protocols, are challenging to detect visually in 2D culture systems. Their accurate identification is essential for ensuring the efficiency and reproducibility of differentiation processes. BrAIn is capable of detecting these structures using the YOLOv8 object detection model,^57^ achieving an initial performance of 67% mean Average Precision (mAP), 62% recall, and 70% precision with a training dataset consisting of 80 images. To enhance detection performance, data augmentation was applied by generating 293 synthetic images from real data using the StyleGAN3 model.^77^ The augmented dataset, comprising a total of 373 images, significantly improved the model's performance, achieving 96% mAP, 94% recall, and 98% precision (Figure 7).

## 4 DISCUSSION

Morphology is one of the key indicators of a healthy and functional organoid. Understanding, monitoring, and controlling organoid morphology is among the focus of leading research groups in the field.^88^, ^89^, ^90^, ^91^ Morphological complexities of BO typically arise from perturbations during developmental phases, resulting in more native‐like brain tissue.^89^ Perturbation can be achieved by carrying out development in different growth conditions^6^, ^8^, ^92^ or by medium modifications.^89^ The 3D nature of BOs enables them to be grown either under static or dynamic culture conditions.^6^, ^76^, ^92^ Orbital shakers and microfluidic chip systems have positive effects on structural and functional properties in BO development and better represent the human brain morphologically.^7^, ^8^ Being able to better analyze BO morphology is quite critical in terms of understanding and improving its functional and structural properties.^89^ However, morphology analyses are quite complex to be done manually and time‐consuming to be done semi‐automatically. In this study, we developed the BrAIn tool that analyzes and quantifies the developmental process of BOs. BrAIn can perform three fundamental computer vision tasks: classification, segmentation, and object detection. It classifies normal, abnormal, and budding morphologies in the transition from EB to BO, quantifies morphological perturbations under diverse culture conditions, performs segmentation to illuminate developmental phases, and detects neural rosette structures. Although other computational tools for organoid morphology exist, BrAIn is the first to combine these tasks under three distinctive culture conditions.

The pluripotency and organoid development abilities of iPSCs in this study have been confirmed in many studies.^75^, ^84^ EBs are 3D cell residues that can differentiate into three different germ layers: ectoderm, endoderm, and mesoderm.^84^, ^93^, ^94^ EBs were confirmed with ectoderm, endoderm, and mesoderm markers, and their differentiation capacity was clarified (Figure S2). Organoids grown in all conditions were phenotypically confirmed for expressing brain tissue characteristics (Figure 2). Many studies have shown that BOs contain SOX2‐expressing neural progenitors and TUJ1‐expressing neurons.^6^, ^76^, ^95^ We confirmed these cells in organoids developed in three different growth conditions. Neural rosettes, formed during ectodermal differentiation, express N‐cadherin, indicating neural cell organization.^96^ These rosettes were verified for structural integrity and neural differentiation (Figure S2).

Early prediction of healthy BOs helps avoid prolonged differentiation of abnormal samples, reducing labor and costs. Although DL models assist wet lab research, studies beyond organoid classification remain limited. DL models are used to identify stem cells,^44^, ^45^, ^46^, ^47^ and while morphological feature‐based classification exists for some organoid types,^48^, ^49^, ^50^, ^51^, ^52^ no such classification has focused on BOs. BrAIn successfully predicted budding–normal and abnormal–normal categories with 92.75% and 96.83% AUC, respectively (Figure 3). It detects target features (Figure 3d) and classifies budding organoids by their budding regions, shown by Grad‐CAM heatmaps. Smoother, more oval shapes are predicted as budding, while sharp boundaries indicate normal samples; in abnormal samples, BrAIn identifies irregular boundaries.

DL models have been more frequently used for segmentation of organoids,^54^, ^55^, ^56^, ^57^, ^58^, ^59^, ^60^, ^61^, ^62^ including neural organoids.^70^, ^71^, ^72^, ^73^, ^74^ Albanese et al. performed automatic 3D ventricle segmentation of cerebral organoids with a U‐Net model (97.2% Dice).^70^ Brémond‐Martin et al. used K‐means and SVM to characterize early BO development^73^ and later adapted U‐Net to achieve 88% Dice.^74^ Deininger et al. segmented BO MRI images (92% Dice)^59^ but did not perform morphology analysis or multi‐class predictions.^72^

BrAIn can segment small 3D cellular structures such as EBs, organoid precursors. EBs are on average 10 times smaller than BOs and range from 50 to 500 μm. BrAIn EBs were segmented with an average IoU of 96.14% and a Dice coefficient of 97.72% and analyzed in terms of average area, Feret diameter, and number (Figure 5a,b). The time‐dependent changes of EBs, which are cell aggregates capable of differentiating into the 3 germ layers that provide the formation of BOs (Figure S2), were examined with BrAIn (Figure 5a). The decrease in the number of EBs from days 1 to 4, when evaluated together with the increase in mean area and ferret diameter, indicates that small cell aggregates merge to form larger ones (Figure 5b). BrAIn segmented BO images collected at 7‐day intervals between days 20 and 62 from organoids cultured under three different growth conditions, including the microfluidic chip system, orbital shaker, and static culture, starting from Day 16. We evaluated the growth conditions in terms of size, geometry, and surface complexity using BrAIn (Figure S5). BrAIn performs BO segmentation with a 98.85% mean IoU and a 98.58% mean Dice coefficient. Our tool evaluates the effect of growth conditions on time‐dependent organoid development with area, Feret diameter, perimeter, roundness, and circularity parameters. Data augmentation for the segmentation part was performed by creating synthetic images with a generative AI model, StyleGAN3 (Figure S4).

We compared the BrAIn U‐Net model with the Swin U‐net,^41^ which includes a transformer model,^36^, ^37^, ^38^ which is another frequently used approach for medical image segmentation. The BrAIn model performed better on BO and EB analysis datasets than the Swin U‐Net (Table S3). This superior performance can be due to the local feature extraction and skip connection structure in small structures in U‐Net.^40^

By segmenting images (Figure 5c) and measuring area, Feret diameter, perimeter, roundness, and circularity (Figure 5), BrAIn reveals how dynamic 3D culture conditions—microfluidic chip and orbital shaker—compare to static culture. Dynamic 3D culture stimulates mechanotransduction, providing a more native‐like microenvironment.^8^, ^9^ We have custom designed a microfluidic system for BOs, supplying continuous shear stress through administrated laminar media flow (Figure S1). Compared to the turbulent flow generated within the orbital shaker and static conditions, the microfluidic chip system environment resulted in distinct clusters of morphological parameters of BOs at all developmental time points of the PCA plot (Figure 5d). While roundness and circularity contributed positively to the largest variance of the PCA plot, size measurements contributed negatively (Figure S5). PCA1 represents the variance of organoids according to their physical size, and we found that dimensional measurements created a certain variance in different growth conditions (Figure S5).

Accordingly, organoids in wells of similar size exhibited parallel growth trends, while those in microfluidic chips showed limited area changes (Figure 5e). Organoids under orbital shaking reached the largest area, possibly due to turbulent flow facilitating nutrient exchange.^6^ Feret diameter and perimeter changes paralleled area differences. The microfluidic chip system displayed more homogeneous, stable growth; circularity and roundness varied more in static and orbital shaker cultures (Figure 5e). BOs grown under orbital shaker and static conditions were maintained in 9.6 cm^2^ (a well of six well plate), whereas the microfluidic chip system offered ~1.1 cm^2^. We previously demonstrated that organoid growth area affects gene expression.^9^ Accordingly, organoids in wells of similar size exhibited parallel growth trends, while those in microfluidic chips showed limited area changes (Figure 5e). BOs under orbital shaking reached the largest area, possibly due to turbulent flow facilitating nutrient exchange.^6^ Feret diameter and perimeter changes paralleled area differences. The microfluidic chip system displayed more homogeneous, stable growth; circularity and roundness varied more in static and orbital shaker cultures (Figure 5e).

To increase generalizability, BrAIn was also evaluated with a different BO dataset (Figure 6). The LAB A subset of the dataset^86^ created by Schröter et al. was segmented by BrAIn, achieving an 84% Dice score, comparable to or better than MOrgAna and OrganoSeg. BrAIn's area‐based measurements closely matched the growth performance reported by Schröter et al., highlighting its applicability to different BO datasets. We also compared BrAIn with the organoid morphology tools OrganoID, OrgaExtractor and SAM (Figures S8 and S9). On our BO dataset, OrganoID had 35% mean IoU and 46% Dice, while OrgaExtractor had 2% IoU and 4% Dice. Additionally, SAM achieved 76.80% IoU and 85.42% Dice coefficient scores. In the EB dataset, OrganoID reached 44% IoU and 58% Dice, and OrgaExtractor 5% IoU and 9% Dice. Furthermore, SAM obtained 31.07% IoU and 4074% Dice coefficient scores. BrAIn scored 98.85% IoU and 98.58% Dice with BOs, and 96.14% IoU and 97.72% Dice with EBs. Although those tools claim general applicability, BrAIn outperformed them on our datasets. Although foundation models such as SAM have broad generalization capabilities, they may have difficulty segmenting layered structures such as organoids and EBs that may have background noise. Organoid and embryoid body images may not have been adequately represented in SAM's large training data. BrAIn may have been more successful in segmentation because it was specifically trained with this data.

BrAIn provides comprehensive analysis for BOs and 2D neuron cultures, but a few limitations remain. Shadows near well‐plate borders can lower segmentation performance in EB images, though dense cell residues did not affect accuracy. Moreover, segmentation of EBs that are in contact with each other is limited. This limitation can be overcome by improving the training dataset to comprise more abundant such images. In addition, the manual segmentation editing feature is considered to be part of BrAIn in future versions, enabling the most ideal segmentation with AI‐based segmentation and custom corrections by the user. Additionally, the number of replicates for morphology analyses at different magnifications is limited, partly due to difficulties in capturing repeated images from complex microfluidic systems.

Published tools focusing on organoid morphology analysis^54^, ^55^, ^56^, ^57^, ^58^, ^59^, ^60^, ^61^, ^62^, ^66^, ^67^, ^68^, ^69^, ^70^, ^71^, ^73^, ^74^ can handle disease modeling and drug effects but are often not user‐friendly for non‐coding users. In contrast, BrAIn has a straightforward interface (Figure S10, Video S1) with its system requirements and installation protocol detailed in Table S4, and its operational software environments outlined in Table S5.

The graphical user interface (GUI) consists of 4 different sections: the main screen, classification, segmentation, and rosette detection. It supports various image formats, including .png, .jpg, and .tif. In the segmentation section, users can input the μm‐per‐pixel conversion factor from their microscope, enabling morphological measurements to be reported in both pixels and micrometers. The segmented organoids are immediately viewable within the GUI. Quantified morphological measurements derived from the segmented images are automatically saved as an Excel file. Also, the detected rosette can be viewed in the GUI, and the coordinates of the rosette on the image can be saved as a text file.

There exist some tools in the literature that perform segmentation^55^, ^56^, ^57^ and object detection^56^, ^68^ tasks on organoids separately. Additionally, there are several morphology analysis tools that are non‐organoid specific and can perform classification, segmentation, and object detection.^97^, ^98^, ^99^, ^100^, ^101^ We summarized the differences between those existing tools and BrAIn in Table S6. Some current organoid morphology analysis tools such as OrganoID have segmentation and object tracking options and offer a user‐friendly interface. However, it cannot classify images and requires a block of code to run, which makes it complicated for non‐coders. MOrgAna and OrgaExtractor both have only segmentation option, making them effective for limited tasks. Whereas OrgaQuant can only perform object tracking and provide GUI. On the other hand, more general morphology analysis tools that are not specific to organoids can be used for morphology analysis for many types of data and can generally perform three different computer vision tasks: classification, segmentation, and object detection (Table S6). Yet, those tools such as CellProfiler may perform constrained segmentation performance for organoids.^84^ SAM, on the other hand, is a foundation segmentation tool providing advanced segmentation options but fails to offer an installation file that can be run with a single click. It requires access over the internet or installation with code to use it. BrAIn does not require an internet connection and is easy to install. Many tools focus on a single task, whereas BrAIn covers classification, segmentation, and object detection, employing widely used models in each domain. With this study, we also present five datasets (Table 3). These BO images—collected across different magnifications and times (days 20–62) using z‐stack methods—encompass early developmental stages and initial maturation. BrAIn and the dataset also provide insight into the effects of different growth conditions on the developmental process of organoids. Unlike general morphology tools, BrAIn is specifically trained to analyze specific features of organoids, such as budding and folding. Compared against the literature, BrAIn has shown acceptable success in analyzing developmental processes of BOs. To our knowledge, BrAIn is the first open‐access tool to combine classification, segmentation, and object detection in a no‐code interface for brain organoid and embryoid body datasets under varying culture conditions.

The BrAIn results may lead to many future studies. In addition to the morphological effects of growth media, omics analyses can be integrated to provide a broader view of how culture conditions influence organoid size and morphology. Different growth media can be evaluated to determine the most optimal conditions for organoid development. Various microfluidic chip designs and flow rates have different effects on organoid development and yield^7^, ^8^; morphological analyses could guide chip design refinements. Since different orbital shaker speeds may affect the development of organoids,^6^, ^89^, ^102^, ^103^ images obtained with BrAIn could help determine the optimum shaker speed.

## 5 CONCLUSION

BrAIn is a powerful DL‐based tool for the automated and objective analysis of BO images. By generating quantitative morphological data and enabling direct comparisons across diverse experimental conditions, it significantly advances BO research and accelerates discoveries in neuroscience and medicine. Moreover, BrAIn's user‐friendly design—requiring no coding expertise—facilitates the comprehensive evaluation of organoid development under various growth conditions, making it a versatile and invaluable asset for studying organoid biology.

## AUTHOR CONTRIBUTIONS

B.K and E.P contributed equally to this work. B.K., E.P. and S.G. established the hypothesis and study design. B.K. and E.P. generated brain organoids, embryoid bodies and neural rosettes. B.K and A.E.E created data sets. B.K. created BrAIn and carried out the computational experiments. B.K. and Y.B. trained AI models. B.K. and H.G trained GAN model and generated synthetic images. All authors contributed the manuscript writing. S.G., Y.B. and G.K edited the manuscript. All the authors read and approved the manuscript. **Sinan Güven**: Conceptualization, methodology, software, data curation, investigation, validation, formal analysis, supervision, funding acquisition, visualization, project administration, resources, writing—original draft, writing—review and editing.

## CONFLICT OF INTEREST STATEMENT

The authors declare no competing financial interest.

## Supporting information


**Data S1.** Supporting information.

**Video S1.** Tutorial for BrAIn.

## Data Availability

The data that support the findings of this study are openly available in Brain Organoid Dataset at https://zenodo.org/records/15513127.
